# *Staphylococcus aureus ftnA* 3’-Untranslated Region Modulates Ferritin Production Facilitating Growth Under Iron Starvation Conditions

**DOI:** 10.3389/fmicb.2022.838042

**Published:** 2022-04-27

**Authors:** Pilar Menendez-Gil, Arancha Catalan-Moreno, Carlos J. Caballero, Alejandro Toledo-Arana

**Affiliations:** Instituto de Agrobiotecnología (IdAB), Consejo Superior de Investigaciones Científicas (CSIC)-Gobierno de Navarra, Navarra, Spain

**Keywords:** *Staphylococcus aureus*, 3’UTRs, post-transcriptional regulation, RNase III, PNPase, mRNA decay, ferritin, iron homeostasis

## Abstract

Iron acquisition and modulation of its intracellular concentration are critical for the development of all living organisms. So far, several proteins have been described to be involved in iron homeostasis. Among them, ferritins act as the major iron storage proteins, sequestering internalized iron and modulating its concentration inside bacterial cells. We previously described that the deletion of the 3’-untranslated region (3’UTR) of the *ftnA* gene, which codes for ferritin in *Staphylococcus aureus*, increased the *ftnA* mRNA and ferritin levels. Here, we show that the ferritin levels are affected by RNase III and PNPase, which target the *ftnA* 3’UTR. Rifampicin mRNA stability experiments revealed that the half-life of the *ftnA* mRNA is affected by both RNase III and the *ftnA* 3’UTR. A transcriptional fusion of the *ftnA* 3’UTR to the *gfp* reporter gene decreased green fluorescent protein (GFP) expression, indicating that the *ftnA* 3’UTR could work as an independent module. Additionally, a chromosomal deletion of the *ftnA* 3’UTR impaired *S. aureus* growth under conditions of iron starvation. Overall, this work highlights the biological relevance of the *ftnA* 3’UTR for iron homeostasis in *S. aureus*.

## Introduction

Iron is an essential micronutrient for several biological processes in living cells such as oxygen transport, methanogenesis, the tricarboxylic cycle (TCA), gene regulation, and DNA biosynthesis (Andrews et al., 2003; Price and Boyd, 2020). When infecting the host, pathogenic bacteria like *Staphylococcus aureus* need to overcome the restricted availability of free iron, a process known as nutritional immunity (Hood and Skaar, 2012; Marchetti et al., 2020). *Staphylococcus aureus*, one of the most relevant nosocomial bacteria worldwide (Tong et al., 2015), has developed diverse strategies to obtain iron by secreting siderophores and hemophores that chelate iron and heme molecules, respectively, as well as expressing iron and heme uptake systems (Wandersman and Delepelaire, 2004; Haley and Skaar, 2012).

Although iron is essential for life, at high cytosolic concentrations, it can be toxic for the bacterium. This is due to the formation of reactive oxygen species (ROS) that cause oxidative stress and damage DNA as well as other essential molecules (Meneghini, 1997; Price and Boyd, 2020). For this reason, bacteria dedicate a significant amount of resources to regulating intracellular iron concentration. In *S. aureus*, the ferric uptake regulator (Fur) and peroxide stress transcriptional regulator (PerR) are the two main transcriptional regulators that modulate the excess of iron and oxidative stress. When high levels of iron are sensed inside the cell, Fur inhibits the transcription of several genes involved in iron and heme uptake and promotes the expression of efflux pumps (Horsburgh et al., 2001; Friedman et al., 2006; Troxell and Hassan, 2013). Alternatively, the iron surplus is dealt with by the storage ferritin protein, FtnA, whose levels are increased under iron-rich conditions. However, when iron is scarce, *ftnA* expression is transcriptionally repressed by PerR (Morrissey et al., 2004; Zühlke et al., 2016).

3’-untranslated regions (3’UTRs) have recently emerged as impactful post-transcriptional regulatory elements that regulate the levels of the mRNAs in which they are encoded through different mechanisms (Miyakoshi et al., 2015; Zhao et al., 2018; Menendez-Gil and Toledo-Arana, 2021). For example, the 3’UTR of the *icaR* mRNA modulates the production of IcaR, the main repressor of PIA-PNAG exopolysaccharide biosynthesis in *S. aureus*. This 3’UTR contains a UCCCC motif that binds to the Shine-Dalgarno (SD) sequence at the 5’UTR of the same mRNA and inhibits ribosome binding and translation. This interaction results in the formation of a double-stranded RNA (dsRNA) substrate for endoribonuclease III (RNase III) to process (de Los Mozos et al., 2013). The 3’UTR of the *Bacillus subtilis hbs* mRNA follows a similar pattern, interacting with its own 5’UTR, but in this case to prevent RNase Y cleavage (Braun et al., 2017). Another way in which 3’UTRs may influence RNA stability and protein expression is by carrying AU-rich motifs that are processed by RNase E and/or PNPase. This has been described for the *Yersinia pestis hmsT*, *Salmonella enterica hilD*, and *Corynebacterium glutamicum aceA* mRNAs, among others (Maeda and Wachi, 2012; López-Garrido et al., 2014; Zhu et al., 2016; Zhao et al., 2018). 3’UTRs can also interact with RNA-binding proteins (RBPs) and small RNAs (sRNAs) to regulate specific biological processes. In *Escherichia coli*, under iron deprivation conditions, the apo-AcnB protein binds to its own mRNA at a loop located in its 3’UTR to protect it against the degradosome (Benjamin and Massé, 2014). The *Salmonella hilD* and *S. aureus icaR* 3’UTRs are targeted by the Spot42 and RsaI sRNAs, respectively, promoting protein expression (El-Mouali et al., 2018; Bronesky et al., 2019). Additionally, 3’UTRs can be reservoirs of *trans*-acting sRNAs that can be generated either by an internal promoter (type I) or by an mRNA processing site (type II) located at the 3’UTR (Miyakoshi et al., 2015).

In a previous study, we unveiled that the *S. aureus ftnA* gene expression was modulated by its own 3’UTR. Deletion of the *ftnA* 3’UTR increased ferritin expression, portraying said 3’UTR as a putative post-transcriptional regulatory element (Menendez-Gil et al., 2020). Here, we show that the *ftnA* 3’UTR could act as an independent regulatory module that is targeted by RNase III and PNPase to decrease ferritin expression. Deletion of the *ftnA* 3’UTR impaired bacterial growth under iron starvation conditions. In this scenario, the *ftnA* 3’UTR-mediated regulation would play an essential role in achieving a tightly regulated iron homeostasis in *S. aureus*.

## Materials and Methods

### Strains, Plasmids, Oligonucleotides, and Growth Conditions

Bacterial strains, plasmids, and oligonucleotides used in this study are listed in Tables 1–3, respectively. *Staphylococcus aureus* strains were grown in Tryptic Soy Broth (Pronadisa) supplemented with 0.25% glucose (TSBg) or, when indicated, in modified chemically defined medium (Toledo-Arana et al., 2005). To prepare *S. aureus* and *E. coli* competent cells, B2 (casein hydrolysate, 10 g L^−1^; yeast extract, 25 g L^−1^; NaCl, 25 g L^−1^; K_2_HPO_4_, 1 g L^−1^; glucose, and 5 g L^−1^; pH 7.5), and SuperBroth (tryptone, 30 g L^−1^; yeast extract, 20 g L^−1^; and MOPS, 10 g L^−1^; pH 7) media were used, respectively. For selective growth, media were supplemented with the appropriate antibiotics at the following concentrations: ampicillin (Amp), 100 μg ml^−1^ for *E. coli* transformants; erythromycin (Erm), 1,5 μg ml^−1^ or 10 μg ml^−1^ for *S. aureus* cells harboring the pMAD or pCN plasmids, respectively.

**Table 1:** Strains used in this study.

Strains	Relevant characteristic(s)	BGR ID^a^	Source or reference
*Staphylococcus aureus*			
15981	MSSA (methicillin sensitive *Staphylococcus aureus*) clinical isolate from an otitis infection; biofilm positive; PIA-PNAG-dependent biofilm matrix.	8	Valle et al., 2003
15981 ∆*ftnA*	15981 carrying a chromosomal deletion of *ftnA* gene.	933	Menendez-Gil et al., 2020
∆*ftnA* p^3xF^FtnA	15981 ∆*ftnA* carrying the p^3xF^FtnA plasmid	1831	Menendez-Gil et al., 2020
∆*ftnA* p^3xF^FtnA∆3’UTR	15,981 ∆*ftnA* carrying the p^3xF^FtnA∆3’UTR plasmid.	1832	Menendez-Gil et al., 2020
15981 p^3xF^FtnA	15981 carrying the p^3xF^FtnA plasmid.	793	This study
15981 p^3xF^FtnA∆3’UTR	15981 carrying the p^3xF^FtnA∆3’UTR plasmid.	794	This study
15981 p^3xF^FtnA∆3’UTR^57-93^	15981 carrying the p^3xF^FtnA∆3’UTR^57-93^ plasmid.	1657	This study
15981 p^3xF^FtnA∆3’UTR^19-56^	15981 carrying the p^3xF^FtnA∆3’UTR^19-56^ plasmid.	2807	This study
15981 pGFP-3UTR*^ftnA^*	15981 carrying the pGFP-3UTR*^ftnA^* plasmid.	1644	This study
15981 pGFP-∆3UTR*^ftnA^*	15981 carrying the pGFP-∆3UTR*^ftnA^* plasmid.	1809	This study
15981 Δ*rnc*	15981 with a deletion of the *rnc* gene.	1760	This study
∆*rnc* p^3xF^FtnA	15981 ∆*rnc* carrying the p^3xF^FtnA plasmid.	1771	This study
∆*rnc* p^3xF^FtnA∆3’UTR	15981 ∆*rnc* carrying the p^3xF^FtnA∆3’UTR plasmid.	1772	This study
∆*rnc* pGFP-3UTR*^ftnA^*	15981 ∆*rnc* carrying the pGFP-3UTR*ftnA* plasmid.	1774	This study
15981 ∆*pnpA*	15981 with a deletion of the *pnpA* gene.	242	Lasa et al., 2011
∆*pnpA* p^3xF^FtnA	15981 ∆*pnpA* carrying the p^3xF^FtnA plasmid.	1628	This study
∆*pnpA* p^3xF^FtnA∆3’UTR	15981 ∆*pnpA* carrying the p^3xF^FtnA∆3’UTR plasmid.	1629	This study
∆*pnpA* pGFP-3UTR*ftnA*	15981 ∆*pnpA* carrying the pGFP-3UTR*ftnA* plasmid.	1646	This study
15981 ∆*rnr*	15981 with a deletion of the *rnr* gene.	243	Lasa et al., 2011
∆*rnr* p^3xF^FtnA	15981 ∆*rnr* carrying the p^3xF^FtnA plasmid.	1630	This study
∆*rnr* p^3xF^FtnA∆3’UTR	15981 ∆*rnr* carrying the p^3xF^FtnA∆3’UTR plasmid.	1631	This study
15981 ∆*mrnc*	15981 with a deletion of the *mrnc* gene.	1762	This study
∆*mrnc* p^3xF^FtnA	15981 ∆*mrnc* carrying the p^3xF^FtnA plasmid.	1777	This study
∆*mrnc* p^3xF^FtnA∆3’UTR	15981 ∆*mrnc* carrying the p^3xF^FtnA∆3’UTR plasmid.	1778	This study
15981 ∆*rny*	15981 with a deletion of the *rny* gene.	1761	This study
∆*rny* p^3xF^FtnA	15981 ∆*rny* carrying the p^3xF^FtnA plasmid.	1783	This study
∆*rny* p^3xF^FtnA∆3’UTR	15981 ∆*rny* carrying the p^3xF^FtnA∆3’UTR plasmid.	1784	This study
15981 ∆*rnjA*	15981 with a deletion of the *rnjA* gene.	1768	This study
∆*rnjA* p^3xF^FtnA	15981 ∆*rnjA* carrying the p^3xF^FtnA plasmid.	1797	This study
∆*rnjA* p^3xF^FtnA∆3’UTR	15981 ∆*rnjA* carrying the p^3xF^FtnA∆3’UTR plasmid.	1798	This study
15981 *ftnA*Δ3’UTR	15981 carrying a deletion of the *ftnA* 3’UTR.	931	This study

a Identification number of the strains stored at the Laboratory of Bacterial Gene Regulation, IdAB-CSIC.

**Table 2:** Plasmids used in this study.

Plasmids	Relevant characteristic(s)	Source and/or reference
pEW	A derivative pCN40 plasmid including the transcriptional terminator region of the pCN47 plasmid downstream of the multiple cloning site.	Menendez-Gil et al., 2020
pAD-cGFP	*Listeria monocytogenes* plasmid carrying the GFP gene with the 5’UTR from *hly* gene under the control of the P*hyper* promoter.	Balestrino et al., 2010
pMAD	*Escherichia coli-Staphylococcus aureus* shuttle vector with a thermosensitive origin of replication for Gram-positive bacteria. The vector contains the *bgaB* gene encoding a β-galactosidase under the control of a constitutive promoter as reporter of plasmid presence. Amp^R^, Erm^R^.	Arnaud et al., 2004
pMAD-∆3’UTR*^ftnA^*	pMAD plasmid containing the allele for deletion of the 3’UTR of *ftnA* gene.	This study
pMAD-∆*rnc*	pMAD plasmid containing the allele for deletion of *rnc* gene.	This study
pMAD-∆*mrnc*	pMAD plasmid containing the allele for deletion of *mrnc* gene.	This study
pMAD-∆*rny*	pMAD plasmid containing the allele for deletion of *rny* gene.	This study
pMAD-∆*rnjA*	pMAD plasmid containing the allele for deletion of *rnjA* gene.	This study
p^3xF^FtnA	pEW plasmid expressing the 3xFLAG-tagged *ftnA* mRNA.	Menendez-Gil et al., 2020
p^3xF^FtnA∆3’UTR	pEW plasmid expressing the 3xFLAG-tagged *ftnA* mRNA lacking the 3’UTR while preserving the transcriptional terminator.	Menendez-Gil et al., 2020
p^3xF^FtnA∆3’UTR^19-56^	pEW plasmid expressing the 3xFLAG-tagged *ftnA* mRNA lacking a region of the 3’UTR including nt 19-56 after the stop codon.	This study
p^3xF^FtnA∆3’UTR^57-93^	pEW plasmid expressing the 3xFLAG-tagged *ftnA* mRNA lacking a region of the 3’UTR including nt 57-93 after the stop codon.	This study
pGFP	pCN40 plasmid expressing GFP with the 5’UTR from *hly* of *L. monocytogenes*.	This study
pGFP-3’UTR*^ftnA^*	pEW plasmid expressing a chimeric mRNA including the *gfp* gene fused to the 3’UTR of *ftnA*.	This study
pGFP-∆3’UTR*^ftnA^*	pEW plasmid expressing a chimeric mRNA including the *gfp* gene fused to the transcriptional terminator of the *ftnA* mRNA.	This study

**Table 3:** Primers used in this study.

Oligonucleotide name	Sequence^a^
**Synthesis of riboprobes**	
NB-probe-3xF-ftn-fw	AATTCATAGTAATTTTAATTTACAA
T7prom-NB-3XF-ftn-rvs	**TAATACGACTCACTATAGGG**GTACTCATGGTTCATTTGATC
NB-probe-ftn-fw	GAAGAACGTTTCCATGGACAAAA
T7-NB-probe-ftn-rvs	**TAATACGACTCACTATAGGG**ACGAGCGCCAAGTTCTTTTTC
**Construction of chromosomic mutant strains**	
D3UTR_ftn_A (BamHI)	*GGATCC*GCAAACTTCTTCATTCAACAAG
D3UTR_ftn_B (NheI)	*GCTAGC*CTGTCTATTGTAGTGATGTTTAAT
Dftn-C (NheI)	*GCTAGC*ACGGAGATCACTAGATTCATTT
Dftn-D (EcoRI)	*GAATTC*GTAGTCAATCCTTTCAATTAATTAAATG
Dftn-E	CAATATCATCAACTTGCTCTG
Dftn-F	CAACATCTTCTGGTTGTATG
Drnc-A (BamHI)	*GGATCC*GGTGAATCGACGTGGAAAAT
Drnc-B (KpnI)	*GGTACC*TTCTAAAACGATTAACTATCTCAC
Drnc-C (KpnI)	*GGTACC*GATTTTAAAACACAATTCCAAGA
Drnc-D (EcoRI)	*GAATTC*AGAACACATGTATACGATATTTTAG
Drnc-E-n	CAGAATTTCTCCCTAAGAAAC
Drnc-F-n	CACCTTTATCGAATTGAACATTG
Dmrnc-A (BamHI)	*GGATCC*CACATTAAATTATTGAATCCATTG
Dmrnc-B (KpnI)	*GGTACC*GCTTCAAAATATCCATTTCTTC
Dmrnc-C (KpnI)	*GGTACC*GAACGATTAGAGGCATTATTAA
Dmrnc-D (EcoRI)	*GAATTC*CTAATTTAGATTTTGGTACAGTTTG
Dmrnc-E	GCAAGGAAAAACAAAGATTTTG
Dmrnc-F	GTACTGTCAATAAACCTTCTT
Drny-A (BamHI)	*GGATCC*CAATAGTTTTATAATCGAGCTTC
Drny_B (KpnI)	*GGTACC*CTCCAACAACTCCTAGAATGATC
Drny-C (KpnI)	*GGTACC*CGATTGGCTAGAGATATTAAAAATC
Drny-D (EcoRI)	*GAATTC*GAAAACCAATCATCTTTATAGGTTTA
Drny-E	CAAATATCCTTATAGGATTGATTG
Drny-F	CTGCAGAAGTTATAAAAGAATTAAAG
DrnjA-A (BamHI)	*GGATCC*GAGTGGGACAGAAATGA
DrnjA-B (KpnI)	*GGTACC*TCAAAAAGCTACTAACTTTGAAGT
DrnjA-C (KpnI)	*GGTACC*TTATTTAGCAATCTCCACATTA
DrnjA-D (EcoRI)	*GAATTC*GATTTAACTGAAATTTTAGTGTTATT
DrnjA-E	CAATTAAACGAGGCAAAGAG
DrnjA-F	CTCATTTAAATTTTACCGTTTCA
pMAD-1	GGAAGCGAGAAGAATCATAATG
pMAD-2	CTAGCTAATGTTACGTTAC
**Construction of plasmids expressing *ftnA* mRNAs**	
+1-ftn (BamHI)	*GGATCC*AATTCATAGTAATTTTAATTTACAA
3’UTR-ftn-19-56-fw	CGAAGAATAATTAAACATCACTACAATACACTTACAATAACCCAATGTCTATATT
3’UTR-ftn-19-56-rv	TATTGTAGTGATGTTTAATTATTCTTCG
3UTR-ftn-term-1/2 (KpnI)	*GGTACC*AAAAAACGCAGATCAATGATTCAGAAAATGAATCTAGTGATCTCCGTGACCCAAATGCCTATCAT
**Construction of plasmids expressing GFP**	
SalI-GFP-fw	*GTCGAC*ATAAAGCAAGCATATAATATTGC
BcuI-TT-BamHI-GFP-rvs	*ACTAGT*AAATGCCTATCCAAGAGGATAGGCATTTTGGATCCTTATTTGTATAGTTCATCCAT
BamHI-EcoRI-3UTR-ftn-fw	*GGATCCGAATTC*TTAAACATCACTACAATAGACAGAT
SmaI-3UTR-ftn-rvs	*CCCGGG*AAAAAACGCAGATCAATGAT
Term ftn (KpnI)	*GGTACC*AAAAAACGCAGATCAATGAT
KpnI-D3UTR-term-ftn	*GGTACC*AAAAAACGCAGATCAATGATTCAGAAAATGAATCTAGTGATCTCCCTGTCTATTGTAGTGATGTTTAAG

a Restriction enzymes sites and T7 promoter sequence are indicated in italic and bold, respectively.

### Plasmid Construction

Most of the plasmids used in this study were engineered as previously described (Caballero et al., 2018; Menendez-Gil et al., 2020). pMAD plasmids used for chromosomal deletions were constructed by amplifying flanking sequences (AB and CD) of the target regions using primers A/B and C/D (Table 3). PCR fragments were digested and ligated into pMAD through a double-fragment ligation process using BamHI, EcoRI, and KpnI or NheI (Tables 2, 3).

The green fluorescent protein (GFP) reporter plasmids were constructed using the *Listeria monocytogenes* pAD-cGFP plasmid as a template (Balestrino et al., 2010). To build pGFP, the *hly* 5’UTR and GFP sequences were amplified with primers Sal-GFP-fw and BcuI-TT-BamHI-GFP-rvs (Table 3) and the resulting PCR fragment was cloned into the pEW plasmid. The 3’UTR of *ftnA* was amplified using primers BamHI-EcoRI-3UTR-ftn-fw and SmaI-3UTR-ftn-rvs (Table 3) and inserted downstream of the *gfp* gene using restriction sites BamHI and SmaI. The pGFP-Δ3’UTR*^ftnA^* was constructed using the pGFP-3’UTR*^ftnA^* as a template and primers SalI-GFP-fw and KpnI-D3UTR-term-ftn (Table 3). The amplification product was ligated into the pGFP plasmid using SalI and KpnI.

The plasmids expressing ^3XF^FtnAΔ3’UTR^19-56^ and ^3XF^FtnAΔ3’UTR^57-93^ were constructed using the p^3xF^FtnA plasmid backbone. For the ^3XF^FtnAΔ3’UTR^19-56^ plasmid, an overlapping PCR was performed using oligonucleotide pairs +1-ftn and 3’UTR-ftn-19-56-fw and 3’UTR-ftn-19-56-rv and term-ftn. Analogously, oligonucleotides +1-ftn and 3UTR-ftn-term-1/2 were used for the ^3XF^FtnAΔ3’UTR^57-93^ plasmid (Table 3). The resulting amplicons were then inserted into pEW using BamHI and KpnI (Table 2).

### Rifampicin mRNA Stability Assay and Northern Blotting

Precultures were grown in 5 ml of TSBg supplemented with Erm (TSBg+Erm) and incubated overnight (ON) at 37°C and 200 rpm. Bacterial concentrations of the precultures were estimated by measuring their optical density (OD_600_). Erlermeyer flasks containing fresh TSBg+Erm were inoculated with precultures to reach starting OD_600_ of 0.02. For rifampicin mRNA stability assays, test cultures were grown at 37°C and 200 rpm until an OD_600_ of 0.5 (exponential phase). Six aliquots of 20 ml of the culture were transferred to 50 ml Falcon tubes containing 300 μg ml^−1^ of rifampicin and incubated at 37°C for 0, 2, 4, 8, 15, and 30 min. Then, 5 ml of stop solution (95% ethanol and 5% phenol) were added to the samples and centrifuged for 2 min at 4,400 *g*. Pellets were frozen in liquid nitrogen and stored at −80°C. RNA extraction and Northern blot analysis were performed as described in Toledo-Arana et al. (2009) and Menendez-Gil et al. (2020). Radiolabeled riboprobes were synthesized from a PCR carrying the T7 promoter (Table 3) using the MAXIscript T7 transcription kit (Ambion) and [α32P]-UTP, following the manufacturer’s recommendations. These riboprobes were designed to target the *ftnA* or the *^3XF^ftnA* mRNAs. The mRNA levels were quantified by densitometry of Northern blot autoradiographies using ImageJ.^1^ Each of the mRNA levels was normalized to the levels of the sample at 0 min of rifampicin incubation.

### Chromosomal Mutagenesis

The mutants generated in this study (Table 1) were obtained as previously described (Valle et al., 2003) by a two-step homologous recombination that exchanges a specific chromosomic region by the mutant allele present in the pMAD plasmid (Arnaud et al., 2004). The marker-less mutants were verified by PCR using oligonucleotides E and F (Table 3) and Sanger sequencing.

### Protein Extraction and Western Blotting

Bacteria were grown as described above. At OD_600_ 0.5, 30 ml samples were taken for protein extraction as described by Menendez-Gil et al. (2020). Western blotting was performed as previously described (Caballero et al., 2018). The 3xFLAG tagged protein samples were incubated with mouse monoclonal anti-FLAG M2-Peroxidase (HRP) antibodies (Sigma) diluted 1:1,000, whereas the GFP samples were incubated with mouse monoclonal anti-GFP antibodies 1:5,000 (Living Colors, Clontech) and peroxidase-conjugated goat anti-mouse immunoglobulin G and M antibodies 1:2,500 (Pierce-Thermo Scientific). Membranes were developed using the SuperSignal West Pico Chemiluminiscent Substrate kit (Thermo Scientific). Mean intensities of developed protein bands were quantified by densitometry of Western blot images using ImageJ and plotted as arbitrary units (A.U.). Statistical significances were calculated by running a paired *t*-test in GraphPad Prim; asterisks (*) indicate *p*-values lower than 0.05 (*p* < 0.05) while *ns* indicate not significant differences.

### Growth Assays Under Iron Limiting Conditions

Precultures were grown in 5 ml of modified chemically defined medium without iron (MM^wo/Fe^) overnight at 37°C and 200 rpm (Toledo-Arana et al., 2005). Since all glass material contains iron traces, bacteria can grow in this medium. In order to eliminate the remaining free iron, we used 2′2-dipyridil (DIP; Sigma) as an iron chelator. Precultures were normalized to OD_600_ 0,1 and 5 μl of these aliquots were diluted in 195 μl of modified chemically defined medium containing different concentrations of DIP in 96-well microtiter plates. The growth curve was monitored using the SpectraMax 340 PC Microplate Reader (Molecular Devices). OD_650_ measurements were performed every 30 min at 37°C for a period of 20 h.

## Results

### Deletion of the *ftnA* 3’UTR Increases the Half-Life of Its mRNA

**Figure 1:**
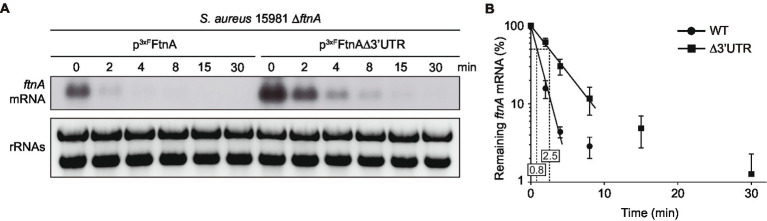
The *ftnA* ∆3’UTR mRNA has a longer half-life than the *ftnA* WT mRNA. **(A)** Half-life measurements of *ftnA* WT and ∆3’UTR mRNAs expressed from a constitutive promoter. Strains carrying the constructs were grown in TSBg at 37°C until exponential phase when 300 μg/ml of rifampicin was added. Samples were taken at the indicated time points (min). Ribosomal RNAs (rRNAs) stained with Midori Green are shown as loading controls. A representative image of the experiment, which was repeated three times, is shown. **(B)** The mRNA levels were quantified by densitometry of Northern blot images using Image J (http://rsbweb.nih.gov/ij/) and normalized using time 0 as a reference. The mean of the mRNA levels was plotted in function of time. Error bars represent the SD from three independent replicates.

In a previous study, we showed that deletion of the *ftnA* 3’UTR increased both the *ftnA* mRNA and ferritin protein levels in *S. aureus* (Menendez-Gil et al., 2020). To evaluate whether the *ftnA* 3’UTR deletion affected *ftnA* mRNA stability, we performed rifampicin mRNA stability assays and half-life determinations. To that end, we transformed the *S. aureus* 15981 Δ*ftnA* strain with the p^3xF^FtnA and p^3xF^FtnAΔ3’UTR plasmids, which expressed the WT and Δ3’UTR *ftnA* mRNAs under the control of the P*_blaZ_* constitutive promoter, respectively. This allowed us to exclusively monitor the plasmidic *ftnA* gene using a strand-specific riboprobe. The resulting strains were grown until exponential phase and their total RNAs extracted at different time points after rifampicin addition. Northern blots revealed that the half-life of the *ftnA* mRNA was higher in the p^3xF^FtnAΔ3’UTR strain (2.5 min) when compared to the p^3xF^FtnA strain (0.8 min; Figure 1). Note that since the decrease in concentration of the two mRNAs was not exponential for all time points, we used only the first three time points (0–4 min) for the WT mRNA and the first four time points (0–8 min) for the Δ3’UTR*ftnA* deletion mutant to calculate their half-life (Figure 1B). These results indicated that the *ftnA* 3’UTR may be targeted by unknown RNases to modulate FtnA expression.

### RNase III and PNPase Target and Process the 3’UTR of the *ftnA* mRNA

**Figure 2:**
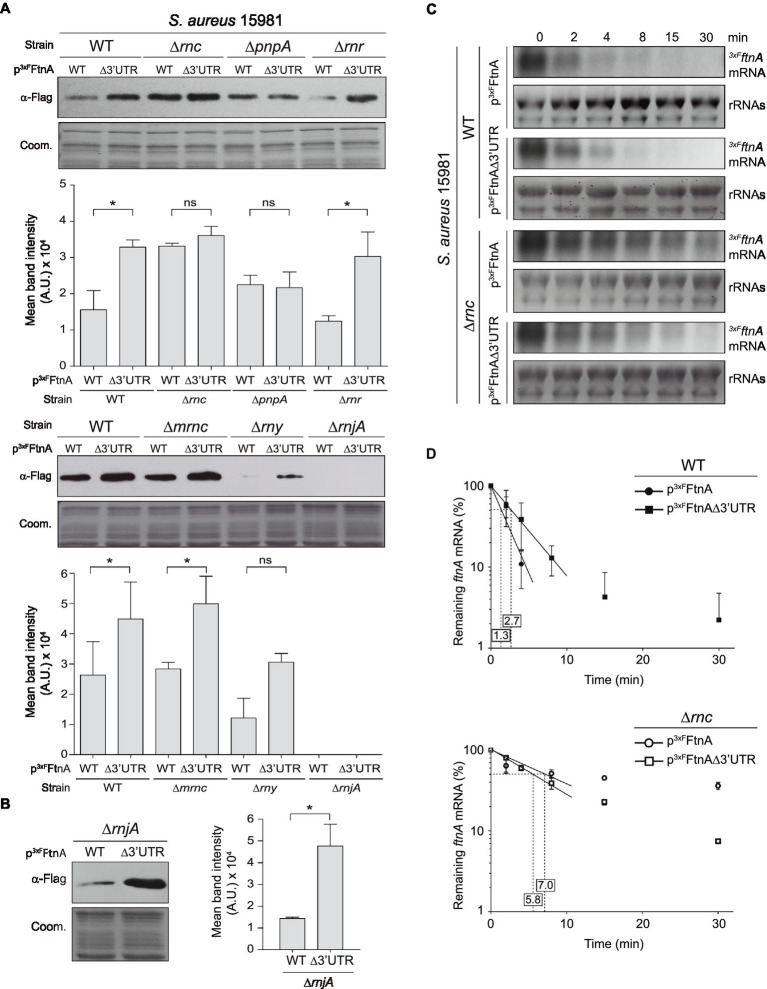
Role of RNases in *ftnA* 3’UTR-mediated regulation. Western blots showing: **(A)** ^3xF^FtnA levels from different *Staphylococcus aureus* 15981 RNase mutants harboring the p^3xF^FtnA and p^3xF^FtnAΔ3’UTR constructs. **(B)** ^3xF^FtnA levels in the ∆*rnjA* mutant increasing the protein load used in **(A)**. Western blots were developed using peroxidase-conjugated anti-FLAG antibodies. Coomassie (Coom.) stained gel portions are shown as loading controls. Western blot images show representative results from at least three independent replicates. The mean intensity of the bands was estimated by densitometry of blot images using ImageJ (A.U., arbitrary units). Statistical significances were determined through paired *t*-tests in GraphPad Prim; ^*^p < 0.05; and ns, not significant. **(C)** Rifampicin half-life assays of 3xFLAG *ftnA* WT and ∆3’UTR mRNAs expressed from a constitutive promoter in the *S. aureus* 15981 WT strain and its isogenic ∆*rnc* mutant. Strains were grown in TSBg at 37°C until exponential phase when 300 μg/ml of rifampicin was added. Samples were taken at the indicated time points (min). rRNAs stained with Midori Green are shown as loading control. Representative images of the experiment, which was repeated twice, are shown. **(D)** The mRNA levels were quantified by densitometry of Northern blot images in Image J (http://rsbweb.nih.gov/ij/) and normalized using time 0 as a reference. The mean of the mRNA levels was plotted in function of time. Error bars represent the SD from two independent replicates.

In order to identify the RNases that could potentially target the *ftnA* 3’UTR, we transformed the most relevant non-essential RNase mutants (Bonnin and Bouloc, 2015) of the *S. aureus* 15981 WT strain with the p^3xF^FtnA and p^3xF^FtnAΔ3’UTR plasmids, which expressed the 3xFLAG-tagged ferritin protein (^3xF^FtnA) from the WT and Δ3’UTR *ftnA* mRNAs, respectively. The selected RNase mutants included ∆*rnc* (RNase III, a dsRNA endonuclease; Lasa et al., 2011; Lioliou et al., 2012), ∆*mrnc* (mini-RNase III, a double-stranded RNA endonuclease paralog to RNase III, which was initially identified in *B. subtilis*; Olmedo and Guzmán, 2008, with an uncharacterized ortholog in *S. aureus*), ∆*pnpA* (PNPase, 3′-5′ exonuclease; Anderson and Dunman, 2009), ∆*rnr* (RNase R, 3′-5′ exonuclease; Oussenko et al., 2002), ∆*rny* (RNase Y, a single-stranded RNA endonuclease; Marincola et al., 2012), and ∆*rnjA* (RNase J1, a bifunctional RNase with endonuclease and 5′ to 3′ exonuclease activities; Linder et al., 2014). We then determined their ^3xF^FtnA protein levels by Western blot using anti-FLAG antibodies (Figure 2A). As expected, the *S. aureus* 15981 WT strain that carried the p^3xF^FtnAΔ3’UTR plasmid expressed higher ^3xF^FtnA protein levels than the strain expressing the whole *ftnA* mRNA. Such protein increase was also obtained when ferritin was expressed from the p^3xF^FtnAΔ3’UTR plasmid in the ∆*rnr*, ∆*mrnc*, and ∆*rny* mutant strains. Since ^3xF^FtnA could not be detected in the ∆*rnjA* strain, we performed the same experiment but loading a higher amount of total protein (Figure 2B). The ∆*rnjA* mutant strains also showed an increase in ferritin expression when the 3’UTR was deleted (Figures 2A,B). These results suggested that RNase R, mini-RNase III, RNase Y, and RNase J1 were not involved in the 3’UTR-mediated processing of the *ftnA* mRNA. In contrast, the Western blots revealed that the ∆*rnc* mutant strains carrying the p^3xF^FtnA and p^3xF^FtnAΔ3’UTR plasmids expressed similar levels of the ^3xF^FtnA protein regardless of the 3’UTR deletion from the 3xFLAG-*ftnA* mRNA. Similar results were obtained in the ∆*pnpA* mutant strains (Figure 2A). This suggests that RNase III and PNPase could be targeting the *ftnA* 3’UTR to process the *ftnA* mRNA.

mRNA decay is often initiated by endoribonucleases, including RNase Y and RNase III (Durand et al., 2015). To confirm the implication of RNase III in the *ftnA* mRNA decay, we evaluated the half-life of the WT and Δ3’UTR*ftnA* mRNAs in *S. aureus* 15981 WT and its isogenic ∆*rnc* mutant strain. The rifampicin mRNA stability assays revealed that the *ftnA* mRNA half-life increased from 1.3 min in the WT strain to 7.0 min in the ∆*rnc* mutant (Figures 2C,D). This was higher than the half-life observed for the Δ3’UTR*ftnA* mRNA mutant, indicating that the RNase III might target the *ftnA* mRNA through additional mechanisms. Note that the half-life of the *ftnA* mRNA and its Δ3’UTR mutant were similar when expressed from the ∆*rnc* mutant (7.0 vs. 5.8 min, respectively; Figures 2C,D). Taken together, these results indicate that RNase III promotes *ftnA* mRNA decay in a process in which the *ftnA* 3’UTR plays a critical role.

### The *ftnA* 3’UTR Works as an Independent Functional Module

**Figure 3:**
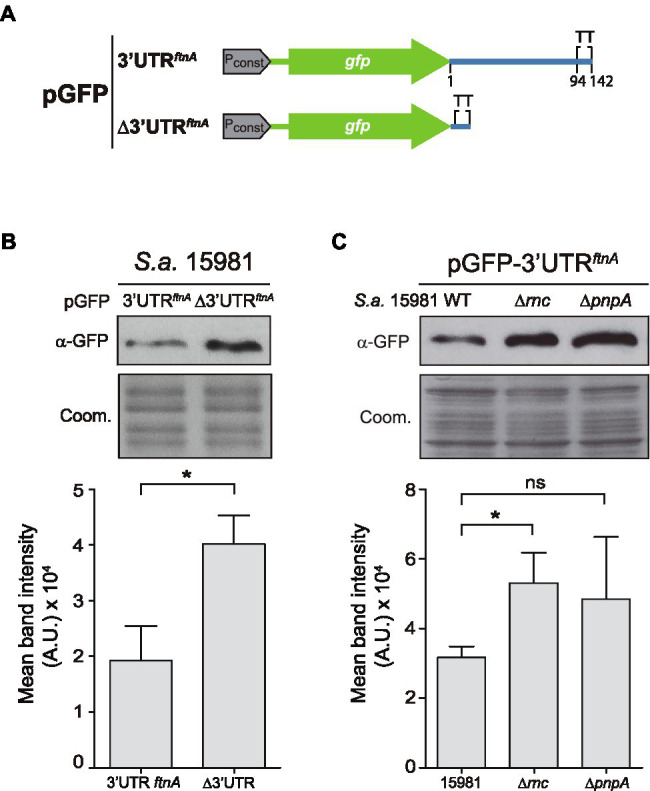
The *ftnA* 3’UTR can act as an independent module. **(A)** Schematic representation of the green fluorescent protein (GFP) constructs generated. P_const_: constitutive promoter; TT: transcriptional terminator. **(B)** Western blot showing the GFP levels of the *Staphylococcus aureus* 15981 WT strain carrying either pGFP-3’UTR*^ftnA^* or pGFP-∆3’UTR*^ftnA^*. **(C)** Western blot showing the GFP levels of the 15981 ∆*rnc* and ∆*pnpA* strains carrying pGFP-3’UTR*ftnA*. Western blots were developed using monoclonal anti-GFP antibodies and peroxidase-conjugated goat anti-mouse immunoglobulin G and M antibodies. Coomassie (Coom.) stained gel portions are shown as loading controls. Western blot images show the representative results from at least three independent replicates. Mean intensity bands were quantified by densitometry of blot images in ImageJ (A.U., arbitrary units). Statistical significances were determined by running paired *t*-tests using the GraphPad Prim software; ^*^p < 0.05; and ns, not significant.

In order to investigate whether the *ftnA* 3’UTR had functional capacities on its own, we fused the *ftnA* 3’UTR downstream of the *gfp* gene, which encodes the GFP, thus, generating the pGFP-3’UTR*^ftnA^* plasmid. As a control, we constructed a plasmid that included the transcriptional terminator (TT) of the *ftnA* mRNA downstream of the *gfp* gene (pGFP-∆3’UTR*^ftnA^*; Figure 3A). Then, we transformed the *S. aureus* 15981 WT strain with these plasmids and determined the GFP levels by Western blot analysis. The results revealed that the pGFP-3’UTR*^ftnA^* plasmid expressed lower GFP levels when compared to the pGFP-∆3’UTR*^ftnA^* plasmid (Figure 3B). This confirmed that the *ftnA* 3’UTR alone worked as an independent module able to reduce the expression of a heterologous gene like *gfp*. To analyze whether such GFP expression reduction was mediated by RNase III and PNPase, we introduced the pGFP-3’UTR*^ftnA^* plasmid into the ∆*rnc* and ∆*pnpA* mutant strains. Western blot results showed that the ∆*rnc* and ∆*pnpA* mutants expressed similar GFP levels to the ones produced by the strain carrying the pGFP-∆3’UTR*^ftnA^* plasmid, indicating that the 3’UTR would still be targeted and processed by RNase III and PNPase regardless of the CDS (Figure 3C).

### The *ftnA* 3’UTR Is Highly Conserved in *Staphylococcus aureus* and *Staphylococcus argenteus*

**Figure 4:**
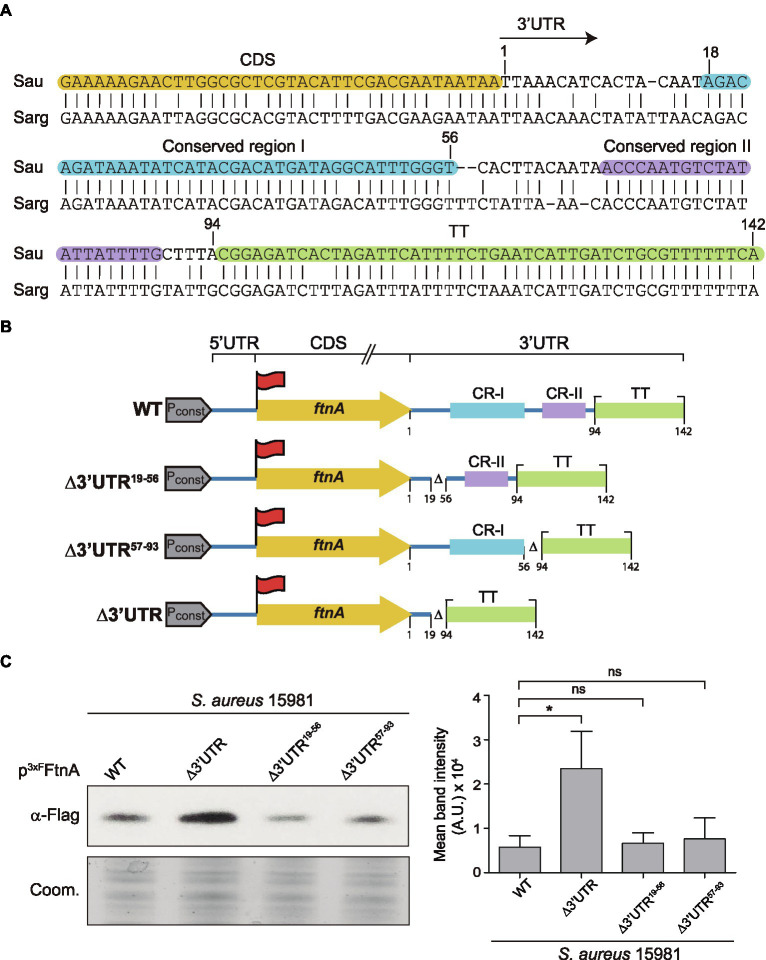
The *ftnA* 3’UTR sequence is conserved in *Staphylococcus aureus and Staphylococcus argenteus*. **(A)** Blastn alignment of the *ftnA* 3’UTR from *Staphylococcus aureus* (Sau) and *Staphylococcus argenteus* (Sarg). Nucleotides corresponding to the CDS, conserved regions I and II (CR-I and CR-II), and the TT are highlighted in orange, blue, purple, and green, respectively. The arrow indicates the start of the 3’UTR. **(B)** Schematic representation of the generated constructs to identify the functional region of the *ftnA* 3’UTR. 3xFlag is represented with a red flag, P_const_: constitutive promoter. **(C)** Western blot showing the levels of ^3xF^FtnA when expressed from the constructs shown in section **(B)**, introduced in the *S. aureus* 15981 WT strain. A Coomassie (Coom.) stained gel portion is shown as a loading control. Western blot images show the representative results from at least three independent replicates. Mean intensity bands were quantified by densitometry of blot images in ImageJ (A.U., arbitrary units). Statistical significances were determined through paired *t*-tests using the GraphPad Prim software; ^*^p < 0.05; and ns, not significant.

Since RNase III is a double-stranded endoribonuclease, we looked for putative double-stranded RNA regions within the *ftnA* 3’UTR secondary structure. We used the RNAstructure version 6.2 software to predict the *ftnA* mRNA conformation.^2^ We could not find any evident secondary structures susceptible to RNase III within the *ftnA* 3’UTR, nor a hypothetical 5’UTR-3’UTR interaction, as previously described for the *icaR* mRNA (de Los Mozos et al., 2013). A plausible alternative would be for the *ftnA* 3’UTR to be targeted by a *trans*-acting small RNA that, upon interaction, generated a double-stranded substrate for RNase III to process. This idea would require the putative paring region to be conserved among *S. aureus* strains and close relatives. Previous multiple sequence alignment analyses revealed that 8,193 out of the 10,000 *S. aureus* genomes available at the NCBI database presented an *ftnA* 3’UTR with a 100% of identity. The remaining ones showed just few nucleotide differences (Menendez-Gil et al., 2020). Although, such a high degree of conservation suggested an important role for the *ftnA* 3’UTR, it prevented us from identifying a putative functional region. Our previous analyses also showed that among other *Staphylococcus* species carrying the *ftnA* gene, the *ftnA* 3’UTR conservation only applied to *S. argenteus* (Menendez-Gil et al., 2020). Further nucleotide comparison analysis between the *S. aureus* and *S. argenteus ftnA* 3’UTRs revealed two conserved regions comprised between nucleotides 18–56 (conserved region I) and 68–89 (conserved region II), respectively, besides the putative TT (Figure 4A). To determine whether one of these regions could be involved in the *ftnA* mRNA processing, we constructed two plasmids expressing the 3xFLAG tagged *ftnA* mRNA carrying either a deletion between nucleotides 19 and 56 (p^3xF^FtnA∆3’UTR^19-56^), or 57 and 93 (p^3xF^FtnA∆3’UTR^57-93^), which selectively eliminated conserved regions I and II, respectively (Figure 4B). We used such plasmids to transform the *S. aureus* 15981 WT strain and evaluated their ^3xF^FtnA protein expression. Western blot analyses revealed that both mutations produced similar ^3xF^FtnA protein levels when compared to the full-length *ftnA* mRNA, suggesting that deletions of a few nucleotides are not enough to reproduce the effect generated by the ∆3’UTR mutant (Figure 4C). At the same time, it indicated that both conserved regions contribute to RNase III action to modulate ferritin expression.

### Deletion of the *ftnA* 3’UTR Impairs *Staphylococcus aureus* Growth During Iron Starvation

**Figure 5:**
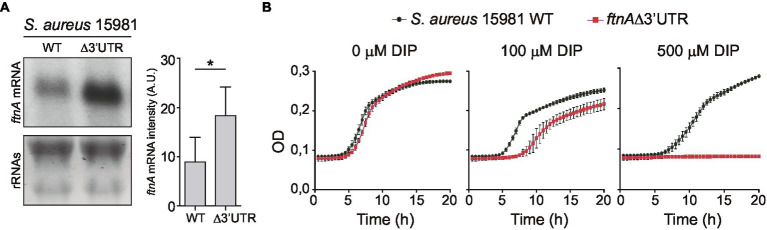
The 3’UTR of *ftnA* is essential for *Staphylococcus aureus* growth under iron starvation conditions. **(A)** Northern blot showing the *ftnA* mRNAs from *S. aureus* 15981 WT and chromosomal *ftnA*Δ3’UTR mutant strains grown in TSB_g_ at 37°C until exponential phase. The lower panel shows ribosomal RNAs (rRNAs) stained with Midori Green as a loading control. A representative image of the experiment, which was repeated twice, is shown. Mean intensity bands were quantified by densitometry of blot images in ImageJ (A.U., arbitrary units). Statistical significances were determined by running paired *t*-tests in GraphPad Prim; ^*^p < 0.05. **(B)** The *S. aureus* 15981 WT and *ftnA*∆3’UTR strains were grown for 20 h in a modified chemically defined medium without iron (MMwo/Fe; Toledo-Arana et al., 2005) and increasing concentrations of the iron chelator 2′2-dipyridil (DIP). Optical density (OD) measurements were registered every half an hour. Error bars represent the SD from three independent replicates.

To evaluate the biological relevance of the *ftnA* 3’UTR-mediated control of ferritin production, we constructed a chromosomal *ftnA*∆3’UTR mutant in the *S. aureus* 15981 genetic background. First, to control that the chromosomal mutant behaved as the plasmidic one, we performed Northern blot analyses to monitor the *ftnA* mRNA levels. Total RNAs were extracted from the WT and ∆3’UTR mutant strains grown until exponential phase in a rich medium (TSBg). Northern blot results showed that the chromosomal *ftnA*∆3’UTR mutant expressed higher *ftnA* mRNA levels than the WT strain as it occurred with the plasmidic mutant (Figure 5A).

Then, we aimed at comparing the capacities of the *S. aureus* 15981 WT and the chromosomal *ftnA*∆3’UTR mutant strains to grow under iron starvation conditions. We incubated microplates containing minimal medium lacking iron (MM^wo/Fe^) at 37°C and measured bacterial growth by registering the optical density every 30 min. However, no growth differences were observed between the WT and ∆3’UTR mutant in the MM^wo/Fe^ (Figure 5B). Since iron traces could be still present in this medium, iron starvation might be difficult to achieve under laboratory conditions without the use of chelating agents (Pi and Helmann, 2017). Therefore, the MM^wo/Fe^ was complemented with increasing concentrations of 2,2′-dipyridyl (DIP), a strong iron chelator. Figure 5B shows that the addition of the DIP chelator at a concentration of 100 μM significantly affected the growth of the *ftnA*∆3’UTR mutant, while adding 500 μM of DIP completely impaired it. Altogether, these data portrayed the *ftnA* 3’UTR as an essential module to control the *ftnA* mRNA expression and maintain proper iron levels for *S. aureus* growth under iron starvation conditions.

## Discussion

The right amount of iron concentration inside the cells is essential for bacterial growth since it is utilized as a cofactor for a wide variety of enzymes. However, intracellular iron excess can lead to oxidative stress and, ultimately, cell damage (Andrews et al., 2003; Hood and Skaar, 2012). For this purpose, the existence of regulating agents such as the ferritin, which removes free intracellular iron, is paramount for protecting cells from its potential toxic effects (Zühlke et al., 2016). As a consequence, the levels of ferritin must also be tightly regulated and in accordance with iron availability (Morrissey et al., 2004). In this study, we showed that ferritin expression is controlled at the post-transcriptional level by the *ftnA* 3’UTR, which is mainly targeted by RNase III and PNPase (Figure 2). The *ftnA* 3’UTR seems to work as an independent *cis*-regulatory module since its fusion to the heterologous *gfp* reporter gene also decreased GFP expression with the participation of RNase III and PNPase (Figure 3). PNPase is a 3′-5′ exoribonuclease whose activity is inhibited by the presence of strong RNA secondary structures (Spickler and Mackie, 2000; Dar and Sorek, 2018; Ingle et al., 2021). The 3′ end of the *ftnA* mRNA contains a putative intrinsic Rho-independent terminator that should avoid PNPase processing. Therefore, one would expect the action of RNase III to trigger *ftnA* mRNA processing, which would provide an mRNA carrying now a 3′ end accessible for PNPase cleavage. This is in agreement with the canonical mechanism of RNA degradation found in the majority of Gram-positive bacteria, which it is initiated by either RNase Y or RNase III and followed by the action of 3′-5′ exoribonucleases such as PNPase and RNase R (Broglia et al., 2020; Mediati et al., 2021). Our data indicated that RNase III would process the *ftnA* mRNA, at least in part, through its 3’UTR (Figure 3). Note that the rifampicin mRNA stability assays also showed that RNase III affects the *ftnA* mRNA independently of the 3’UTR (Figure 2). This mechanism would require further investigations.

RNase III cleaves dsRNAs. However, no internal double-stranded RNA structures were predicted in the 3’UTR that could provide a dsRNA substrate for RNase III as previously described (de Los Mozos et al., 2013). This suggested that either a *cis*-antisense RNA or a *trans*-acting sRNA may be required to create such RNA substrate for RNase III to cleave (Lasa et al., 2011; Lioliou et al., 2012). Although, we predicted some putative interactions between the *ftnA* 3’UTR and previously identified sRNAs in *S. aureus* (Geissmann et al., 2009; Bohn et al., 2010; Carroll et al., 2016), we failed to validate such interactions *in vivo* (data not shown). Whether other sRNAs and/or asRNAs interact with the *ftnA* 3’UTR remains to be explored. Moreover, knowing that global regulatory RNA chaperones have been already shown to bind 3’UTRs (Holmqvist et al., 2016, 2018; Potts et al., 2017) and iron-sensing proteins like aconitase bind their own mRNA (Benjamin and Massé, 2014), it would be interesting to evaluate whether RNA-binding proteins or even the ferritin itself could interact with the *ftnA* mRNA through the 3’UTR to control ferritin expression.

**Figure 6:**
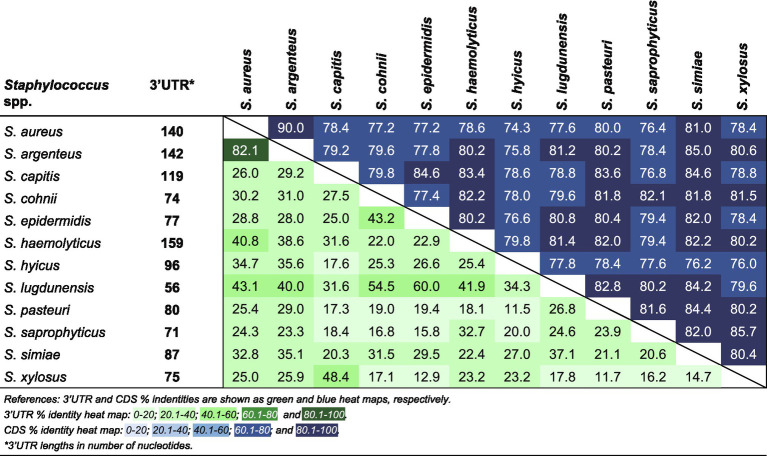
Percent identity matrix of a multiple sequence alignment for the *ftnA* 3’UTRs and *ftnA* CDSs from several *Staphylococcus* species.

It is noteworthy that the whole *ftnA* 3’UTR was highly conserved in *S. aureus* and *S. argenteus*. This suggested that the *ftnA* 3’UTR sequence may be relevant for both species, which preserve throughout evolution a similar post-transcriptional control of ferritin production. It is also interesting that the corresponding 3’UTR sequences from other *Staphylococcus* species were completely different (both in length and sequence) despite the *ftnA* CDS being conserved (Figure 6). We previously showed that constructs of chimeric mRNAs including the *S. aureus ftnA* CDS and the *ftnA* 3’UTRs from *Staphylococcus simiae*, *Staphylococcus epidermidis*, and *Staphylococcus capitis* were unable to decrease ^3xF^FtnA expression (Menendez-Gil et al., 2020), indicating the presence of species-specific 3’UTR-mediated regulatory mechanisms. How the *ftnA* 3’UTRs from different *Staphylococcus* species participate in the modulation of ferritin production remains to be investigated.

These putative regulatory differences are not restricted to the post-transcriptional level. It was also shown that the transcriptional regulation of ferritin expression in response to metals in *S. epidermidis* was significantly different from *S. aureus* (Morrissey et al., 2004), suggesting that members of the *Staphylococcus* genus have developed different strategies to regulate iron homeostasis in species-specific manners.

In addition to the *ftnA* 3’UTR, we recently found 3’UTR sequence variability in several staphylococcal genes, indicating that this phenomenon may be widespread among bacteria. Asides from iron homeostasis, long 3’UTRs with evolutionary variability (Menendez-Gil et al., 2020) also affect relevant biological processes such as metabolism (Maeda and Wachi, 2012), biofilm formation (de Los Mozos et al., 2013; Zhu et al., 2016), and hemolysin production (Menendez-Gil et al., 2020). We proposed that these regions may be prone to changes that reflect in bacterial diversity in a similar way as it occurred for eukaryotes, promoting the diversification of species (Menendez-Gil and Toledo-Arana, 2021).

Another relevant observation in this study was the impaired growth of *S. aureus* under iron starvation conditions upon chromosomal deletion of the *ftnA* 3’UTR (Figure 5). Considering that deletion of the *ftnA* 3’UTR increased ferritin concentration (Figure 2; Menendez-Gil et al., 2020), it could be speculated that higher ferritin levels would sequester the scarce iron available inside the cells. As a result, the essential functions carried out by enzymes requiring iron as a cofactor would be affected, leading to bacterial growth arrest.

In summary, our study highlights the relevance of 3’UTRs to fine-tune the expression of genes involved in relevant processes such as iron homeostasis.

## Data Availability Statement

The original contributions presented in the study are included in the article, further inquiries can be directed to the corresponding author.

## Author Contributions

PM-G and AT-A conceived and designed the experiments. PM-G and AC-M performed the experiments. PM-G, AC-M, CC, and AT-A analyzed the data and contributed to the interpretation of results. PM-G, CC, and AT-A wrote the manuscript. All authors contributed to the article and approved the submitted version.

## Funding

AT-A was supported by the European Research Council under the European Union’s Horizon 2020 research and innovation program (ERC-CoG-2014-646869); and the Spanish Ministry of Science and Innovation (PID2019-105216GB-I00) grants.

## Conflict of Interest

The authors declare that the research was conducted in the absence of any commercial or financial relationships that could be construed as a potential conflict of interest.

## Publisher’s Note

All claims expressed in this article are solely those of the authors and do not necessarily represent those of their affiliated organizations, or those of the publisher, the editors and the reviewers. Any product that may be evaluated in this article, or claim that may be made by its manufacturer, is not guaranteed or endorsed by the publisher.

## Footnotes

^1^ http://rsbweb.nih.gov/ij/

^2^ http://rna.urmc.rochester.edu/RNAstructure.html
